# Short-term and long-term prognostic value of circulating soluble suppression of tumorigenicity-2 concentration in acute coronary syndrome: a meta-analysis

**DOI:** 10.1042/BSR20182441

**Published:** 2019-06-04

**Authors:** Linlin Gu, Jing Li

**Affiliations:** Department of Emergency Intensive Care Unit, Affiliated Hospital of Jining Medical College, Jining 272200, China

**Keywords:** acute coronary syndrome, meta-analysis, prognosis, soluble suppression of tumorigenicity-2

## Abstract

**Background:** Higher circulating soluble suppression of tumorigenicity-2 (sST2) concentration is suggested as a marker of prognosis in many cardiovascular diseases. However, the short-term and long-term prognostic value of sST2 concentration in acute coronary syndrome (ACS) remains to be summarized.

**Methods:** A meta-analysis of follow-up studies was performed. Studies were identified via systematic search of databases including PubMed, Cochrane’s Library, and Embase. A fixed- or random-effect model was applied according to the heterogeneity. We reported the prognostic value of sST2 concentration for all-cause mortality, heart failure (HF) events, and major adverse cardiovascular events (MACEs) within 1 month after hospitalization and during subsequent follow-up.

**Results:** Twelve studies with 11690 ACS patients were included. Higher baseline sST2 concentration as continuous variables predicte the increased risk of all-cause mortality (risk ratio [RR]: 3.16, *P*=0.002), HF events (RR: 1.48, *P*<0.001), and MACEs (RR: 1.47, *P*<0.001) within 1 month after hospitalization, which is consistent with the results with sST2 concentration as categorized variables (RR = 2.14, 2.89, and 2.89 respectively, *P* all <0.001). Moreover, higher baseline sST2 concentration as continuous variables predict the increased risk of all-cause mortality (RR: 2.20, *P*<0.001), HF events (RR: 1.39, *P*<0.001), and MACEs (RR: 1.53, *P*=0.02) during subsequent follow-up. Meta-analysis with sST2 concentration as categorized variables retrieved similar results (RR = 2.65, 2.59, and 1.81 respectively, *P* all <0.001).

**Conclusions:** Higher circulating sST2 concentration at baseline predicts poor clinical outcome in ACS patients.

## Introduction

With the aging of the global population, the prevalence of coronary artery disease (CAD) is increasing. Acute coronary syndrome (ACS), including ST segment elevated myocardial infarction (STEMI) and non-ST segment elevated ACS (NSTE-ACS) has now become the leading cause of mortality in general population, particularly in the elderly [1,2]. It was reported that approximately 800000 people experienced ACS annually in the United States, and approximately 30% of them had STEMI [3,4]. Characterized by acute plaque rupture and thrombosis formation in the coronary arteries, patients with ACS usually have higher risk for the development of heart failure (HF) and death [4–6]. Biomarkers such as cardiac troponin and B-type natriuretic peptide (BNP) play important roles for risk estimation in ACS patients [7,8]. Besides, recent studies indicate that other biomarkers such as soluble suppression of tumorigenicity-2 (sST2) concentration also confer prognostic value in patients with ACS [9,10]. Pathophysiologically, interleukin 33 (IL-33) mediates various potential cardioprotective effect via interaction with transmembrane ST2 (ST2L), including anti-inflammation, anti-remodeling, and hypertrophy, and anti-apoptosis. Therefore, the sST2 has been demonstrated to attenuate the potential cardioprotective effect of IL-33/ST2L via acting as a decoy receptor [11]. Accordingly, previous meta-analyses showed that higher circulating sST2 concentration is associated with worse clinical outcomes in patients with acute and chronic HF [12,13]. For patients with ACS, although most of the pilot follow-up studies suggested that higher sST2 concentration at baseline predicts increased mortality risk [14–25], these studies vary in scales and follow-up durations, and quantitative analyses for the prognostic efficacy of sST2 concentration at baseline for short-term and long-term outcomes in ACS patients have not been performed. Therefore, the aim of the meta-analysis was to summarize the potential prognostic efficacy of sST2 concentration for short-term and long-term outcomes in ACS patients.

## Methods

### Database search

We performed this meta-analysis as instructed by the MOOSE (Meta-analysis of Observational Studies in Epidemiology) [26] and Cochrane’s Handbook [27] guidelines. Databases of PubMed, Cochrane’s Library, and Embase were searched for potential studies with the combined search terms of ‘suppression of tumorigenecity–2’, ‘suppression of tumorigenicity–2’, ST2, sST2), and ‘myocardial infarction’, ‘acute coronary syndrome’, ‘ACS’, ‘unstable angina’, STEMI, or NSTEMI, on 29 October 2018. The search was limited to studies in humans that were published in English. The references of the related original papers and review articles were manually searched for additional potential studies.

### Inclusion and exclusion criteria

Studies fulfilling the following criteria were included: (i) follow-up studies, including post-hoc analysis of randomized controlled trials; (ii) included patients with ACS; (iii) sST2 concentration was measured at baseline; (iv) reported the adjusted risk ratios (RRs) and their corresponding 95% confidence intervals (CIs) for the incidences of all-cause mortality, HF events (HF incidence or hospitalization), and major adverse cardiovascular events (MACEs). Definition of MACEs was inconsistent with the original studies, including cardiovascular death, HF incidence or worsening, recurrent MI, and repeated target vessel revascularization (TVR). Reviews, meta-analysis, preclinical studies, and non-follow-up studies were excluded.

### Data extraction and quality evaluation

The following data were extracted: the study design, diagnosis of the patients, sample sizes, mean ages, proportions of males, methods for measuring sST2 concentration, follow-up durations, variables adjusted, and the statistical presentation of sST2 concentration (as continuous or categorized variables). The quality evaluation was performed with the Newcastle–Ottawa Scale [28] which ranges from 1 to 9 stars and evaluates the quality of each study based on three aspects: selection of the study groups; the comparability of the groups; and the ascertainment of the outcome of interest. Two reviewers performed database search, data extraction, and quality evaluation independently.

### Statistical analyses

We used RRs as the general measure for the prognostic efficacy of sST2 concentration for ACS. For studies presenting sST2 concentration as continuous variables, log transformation of sST2 concentration was performed in the original studies because of the skewed distribution, and RRs for log (sST2) were extracted. For those sST2 concentration was presented as categorized variables, RRs for comparing patients form the highest and the lowest category of sST2 concentration were extracted. We calculated corresponding stand errors (SEs) or RRs from 95% CIs or *P* values, and logarithmically transformed them to stabilize the variance and normalize the distribution [27]. The Cochrane’s Q test and *I^2^* test were used to evaluate the heterogeneity among the include cohort studies [27,29]. A significant heterogeneity was considered if *I^2^* > 50%. We used a random-effect model to synthesize the RR data if heterogeneity was significant; otherwise, a fixed-effect model was applied. We reported the prognostic value of sST2 concentration for each outcome both within 1 month after hospitalization and during subsequent follow-up. Potential publication bias was evaluated by funnel plots with the Egger regression asymmetry test [30]. The RevMan (Version 5.1; Cochrane Collaboration, Oxford, U.K.) and STATA software were applied for the statistics.

## Results

### Search, study inclusion, and characteristics

**Figure 1:**
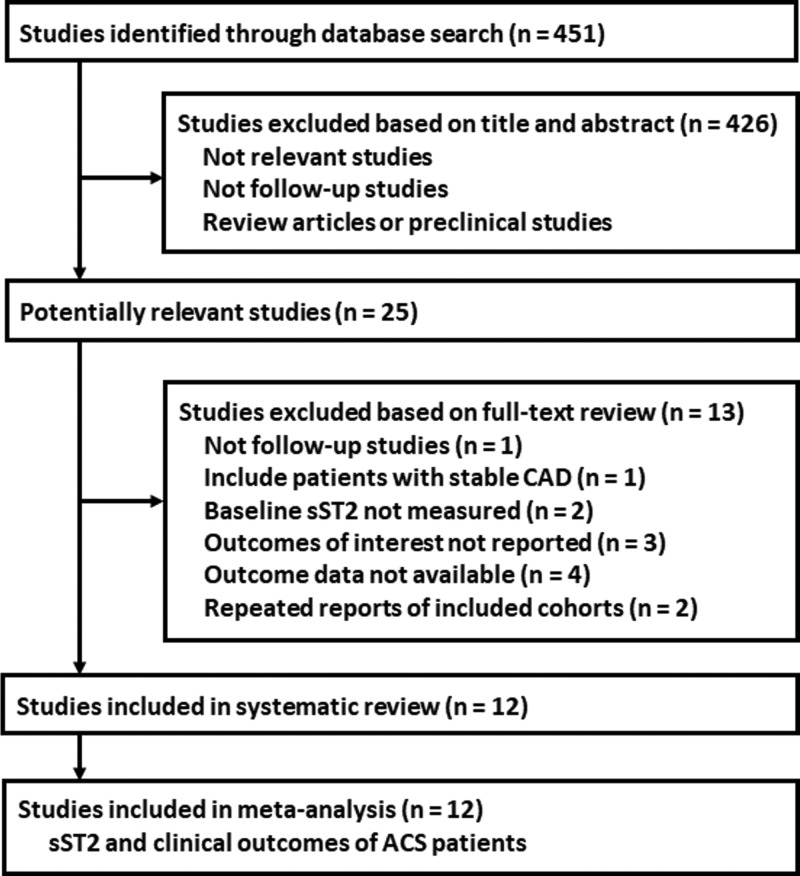
Process of database search and study inclusion

The flowchart of database search is presented in Figure 1. Of the 451 initially identified studies, 12 were finally included [14–25] and listed in Table 1. This meta-analysis included five post-hoc analysis [14–16,18,20] and seven prospective cohort studies [17,19,21–25] with 11690 ACS patients. Eight studies included STEMI patients [14,15,19,20,22–25], three studies included NSTE-ACS patients [16–18], and the other one included both [21]. Baseline circulating sST2 concentrations were measured with enzyme-linked immunosorbent assay (ELISA) methods from MBL, R&D, and Presage, while rapid test was applied in one study [23]. The follow-up varied from 1 month to 5 years. Various confounding factors such as age, gender, medical histories, comorbidities, biochemical parameters, and treatments were adjusted when presenting the RRs in the included studies. The NOS varied from 7 to 9 points, indicating generally good study qualities.

**Table 1:** Characteristics of the included cohort studies

Study	Country	Design	Diagnosis	Sample size	Age	Male	sST2 measurement	Follow-up time	sST2 cutoff	Variables adjusted	NOS
					Years	%		Months			
Shimpo 2004 [14]	U.S.A.	Post-hoc	STEMI	810	58	80	MBL ELISA assay	1	Continuous	Age, HR, SBP, infarct location, Killip class, time from symptom onset, and TIMI flow grade of IRA	7
Sabatine 2008 [15]	U.S.A.	Post-hoc	STEMI	1239	58	78	MBL ELISA assay	1	Continuous and Q4/Q1	Age, sex, hypertension, DM, prior MI, prior CHF, eGFR, infarct location, Killip class, time from symptom onset, and peak CK	7
Eggers 2010 [16]	Sweden	Post-hoc	NSTE-ACS	403	69	65	Presage ST2 assay	12	Continuous	Age, CHF, DM, previous MI, and previous stroke	8
Dhillon 2011 [17]	U.K.	PC	NSTEMI	577	70	69	ELISA (R&D)	1 and 18	Continuous	Age, gender, smoking previous angina or AMI, HF, hypertension, DM, Killip class, eGFR, FBG, TnI, use of BBs and statins	8
Kohli 2012 [18]	U.S.A.	Post-hoc	NSTE-ACS	4426	NA	66	Presage ST2 assay	1 and 12	Continuous and Q4/Q1-3	Age, CAD, DM, hypertension, dyslipidemia, severe angina, ST changes, smoking, history of HF, eGFR, TnI, BNP, hsCRP, and use of aspirin	8
Dhillon 2013 [19]	U.K.	PC	STEMI	677	64	75	ELISA (R&D)	1 and 12	Continuous	Age, gender, previous history of angina/AMI, hypertension, DM, Killip Class, eGFR, peak CK, treatment with thrombolysis, BB, statins, ACEIs or ARBs	9
O’Donoghue 2016 [20]	U.S.A.	Post-hoc	STEMI	1258	58	79	MBL ELISA assay	1	Q4/Q1-3	Age, sex, past HF, DM, past MI, SBP, HR, Killip class, infarct location, eGFR, and time from symptom onset	7
Jenkins 2017 [21]	U.S.A.	PC	AMI (STEMI: 291, NSTEMI: 1110)	1401	67	61	Presage ST2 assay	1, 12 and 60	Continuous and T3/T1	Age, sex, Charlson comorbidity index, Killip class, and maximum TnT	9
Yu 2017 [22]	Korea	PC	STEMI	323	59	84	ELISA (R&D)	12	Median	Age, DM, final TIMI flow grade, hypoxic liver injury, hs-CRP level, and TnI level	8
Huang 2018 [24]	China	PC	STEMI	186	62	74	Presage ST2 assay	12	Median	Age, gender, smoking, SBP, HR, Killip Class, LVEF, eGFR, NT-proBNP, TNI, CRP, and pPCI	8
Hartopo 2018 [23]	Indonesia	PC	STEMI	95	58	76	ASPECT PLUS Rapid ST2 Test	12	Median	Age, DM, HR, Hb, SCr, FBG, TG, TnI, and infarct location	8
Liu 2018 [25]	China	PC	STEMI	295	60	83	Presage ST2 assay	12	Q4/Q1	Age, gender, smoking, SBP, HR, Killip Class, LVEF, eGFR, infarct location, time from onset to ER, NT-proBNP, TNI, CRP, and IL-6	8

Abbreviations: ACEI, angiotensin converting enzyme inhibitor; AMI, acute myocardial infarction; ARB, angiotensin II receptor blocker; BB, β blocker; CHF, congestive HF; CK, creatine kinase; DM, diabetes mellitus; eGFR, estimated glomerular filtrating rate; ER, emergency room; FBG, fasting blood glucose; Hb, hemoglobin; HR, heart rate; hs-CRP, high-sensitive C-reactive protein; IL-6, interleukin 6; IRA, infarct related artery; LVEF, left ventricular ejection fraction; MBL, Medical & Biological Laboratories; MI, myocardial infarction; NOS, Newcastle–Ottawa Scale; PC, prospective cohort; NT-proBNP, N-terminal pro BNP; pPCI, primary percutaneous coronary intervention; Q, quartile; SBP, systolic blood pressure; T, tertile; TG, TIMI, Thrombolysis in Myocardial Infarction; triglyceride; TnI, troponin I; TnT, troponin T.

### Short-term prognostic value of sST2 concentration in ACS

**Figure 2:**
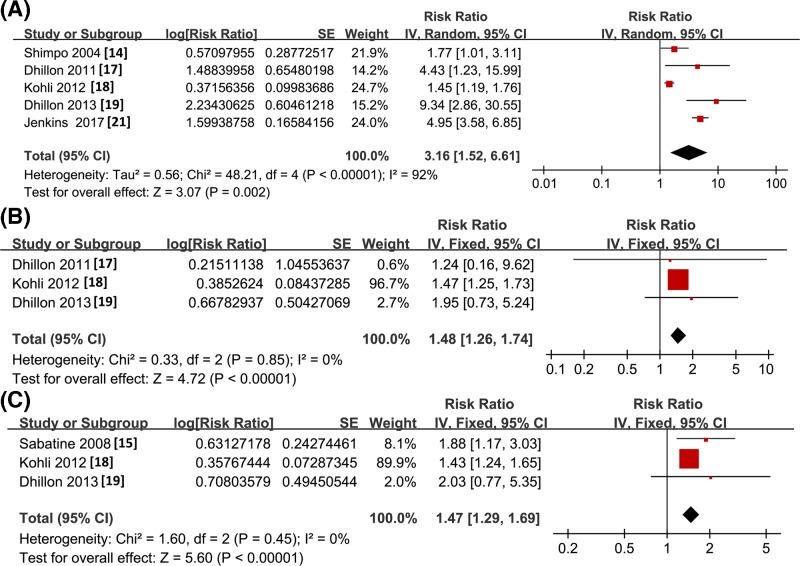
Forest plots for the meta-analysis of short-term prognostic value of sST2 concentration in ACS patients with sST2 concentration presented as continuous variable (**A**) All-cause mortality; (**B**) HF events; (**C**) MACEs.

**Figure 3:**
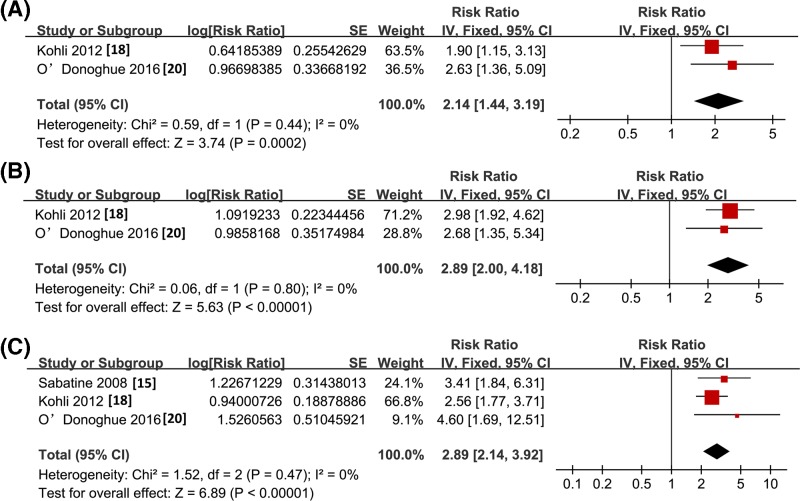
Forest plots for the meta-analysis of short-term prognostic value of sST2 concentration in ACS patients with sST2 concentration presented as categorized variable (**A**) All-cause mortality; (**B**) HF events; (**C**) MACEs.

Pooled results with three to five studies showed that higher baseline sST2 concentration as continuous variables predict the increased risk of all-cause mortality (RR: 3.16, 95% CI: 1.52–6.61, *P*=0.002; *I^2^* = 92%; Figure 2A), HF events (RR: 1.48, 95% CI: 1.26–1.74, *P*<0.001; *I^2^* = 0%; Figure 2B), and MACEs (RR: 1.47, 95% CI: 1.29–1.69, *P*<0.001; *I^2^*= 0%; Figure 2C) within 1 month after hospitalization. These results were further confirmed by meta-analysis of studies with sST2 concentration presented as categorized variables. Compared with patients with baseline sST2 concentration in the lowest categories, those in the highest categories had significantly higher incidences of short-term all-cause mortality (RR: 2.14, 95% CI: 1.44–3.19, *P*<0.001; *I^2^* = 0%; Figure 3A), HF events (RR: 2.89, 95% CI: 2.00–4.18, *P*<0.001; *I^2^* = 0%; Figure 3B), and MACEs (RR: 2.89, 95% CI: 2.14–3.92, *P*<0.001; *I^2^* = 0%; Figure 3C). The publication biases of the above meta-analyses were difficult to estimate due to the limited number of studies included.

### Long-term prognostic value of sST2 concentration in ACS

**Figure 4:**
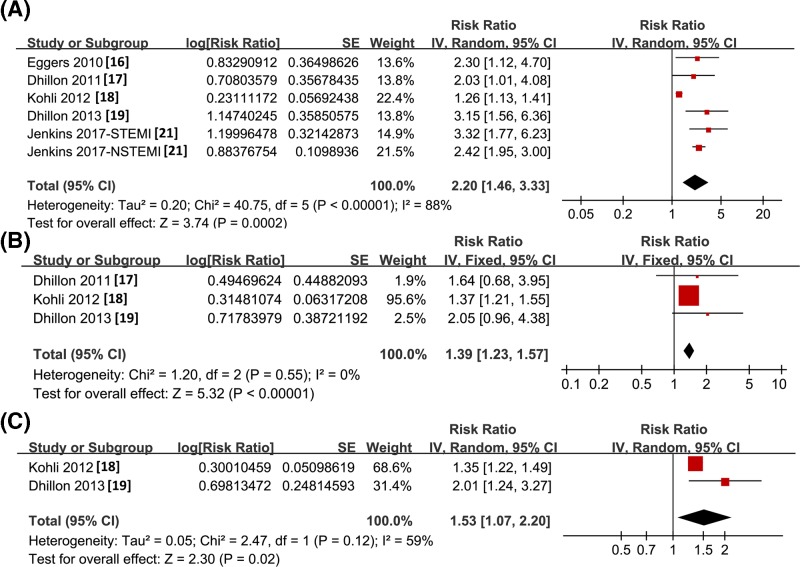
Forest plots for the meta-analysis of long-term prognostic value of sST2 concentration in ACS patients with sST2 concentration presented as continuous variable (**A**) All-cause mortality; (**B**) HF events; (**C**) MACEs.

**Figure 5:**
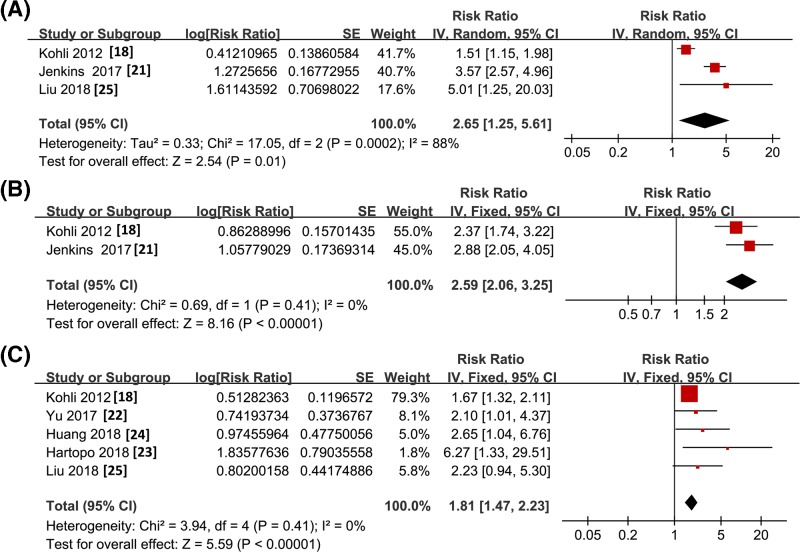
Forest plots for the meta-analysis of long-term prognostic value of sST2 concentration in ACS patients with sST2 concentration presented as categorized variable (**A**) All-cause mortality; (**B**) HF events; (**C**) MACEs.

Pooled results with two to six studies showed that higher baseline sST2 concentration as continuous variables predict the increased risk of all-cause mortality (RR: 2.20, 95% CI: 1.46–3.33, *P*<0.001; *I^2^* = 88%; Figure 4A), HF events (RR: 1.39, 95% CI: 1.23–1.57, *P*<0.001; *I^2^* = 0%; Figure 4B), and MACEs (RR: 1.53, 95% CI: 1.07–2.20, *P*=0.02; *I^2^* = 59%; Figure 4C) during subsequent follow-up to 5 years after hospitalization. These were further confirmed by meta-analysis with sST2 concentration as categorized variables (all-cause mortality: RR: 2.65, 95% CI: 1.25–5.61, *P*<0.001; *I^2^* = 88%; Figure 5A; HF events: RR: 2.59, 95% CI: 2.06–3.25, *P*<0.001; *I^2^* = 0%; Figure 5B; and MACEs: RR: 1.81, 95% CI: 1.47–2.23, *P*<0.001; *I^2^* = 0%; Figure 5C). The publication biases of the meta-analyses could not be estimated due to the limited number of included studies.

### Sensitivity analyses

In view of the fact that the patients included in the study by Kohli et al. [18] accounted for 37.86% of the whole patients of the whole meta-analysis, sensitive analyses by omitting this study were performed. The results were not changed for most of the outcomes after omitting the study by Kohli et al. [18] except that the prognostic efficacy of sST2 concentration as continuous variable for short-term HF events becomes insignificant (RR: 1.79, 95% CI: 0.74–4.36, *P*=0.20; Table 2).

**Table 2:** Sensitivity analyses by omitting the study by Kohli et al. [18]

Outcomes	Number of studies	RR (95% CI)	*P*-values
Short-term			
All-cause mortality (continuous)	4	3.99 [1.99, 8.02]	<0.001
HF events (continuous)	2	1.79 [0.74, 4.36]	0.20
MACEs events (continuous)	2	1.91 [1.24, 2.92]	0.003
All-cause mortality (categories)	1	2.63 [1.36, 5.09]	0.004
HF events (categories)	1	2.68 [1.35, 5.34]	0.005
MACEs events (categories)	2	3.70 [2.19, 6.26]	<0.001
Long-term			
All-cause mortality (continuous)	5	2.49 [2.08, 2.99]	<0.001
HF events (continuous)	2	1.86 [1.05, 3.31]	0.03
MACEs events (continuous)	1	2.01 [1.24, 3.27]	0.005
All-cause mortality (categories)	2	3.64 [2.64, 5.01]	<0.001
HF events (categories)	1	2.88 [2.05, 4.05]	<0.001
MACEs events (categories)	4	2.49 [1.57, 3.93]	<0.001

## Discussion

Current risk stratification for patients with STEMI and NSTE-ACS mainly depends on the application of risk stratification systems including TIMI (Thrombolysis in Myocardial Infarction) [31] and GRACE (Global Registry of Acute Cardiac Events) [32] risk scores. As these systems have been proved to confer satisfying efficacies for risk stratification and recommended by current guidelines for ACS, it has been decades since the validation of the risk scores, and changes of the disease profiles and treatment patterns may require adding new factors to optimize the prognostic efficacies of these tools. Novel cardiac biomarkers such as sST2 may be one of them [9]. In this study, based on the meta-analysis of multivariable adjusted follow-up studies, demonstrated that baseline level of sST2 concentration is an important prognostic factor for the clinical outcomes in ACS, including all-cause mortality, HF events, and MACEs. The independent association between circulating sST2 concentration and poor clinical outcomes in ACS was observed after the full adjustment of potential confounding factors in our meta-analysis, which was independent of the follow-up durations and patterns of sST2 concentration presentation in statistical analyses. Our results, therefore, it can be estimated that including circulating sST2 concentration into the above ACS risk sores may improve their overall predictive efficacy. In fact, a recent study including MI patients showed that incorporation of sST2 concentration into the GRACE and TIMI risk scores significantly improved the discriminatory performances of the systems [33]. Moreover, as a cardiac biomarker, sST2 concentration in circulation is found to be stable and unlikely to be affected by age, body mass index, or renal function [34–37]. These findings support that incorporation of measuring sST2 concentration is rationale for risk stratification and clinical decision making for ACS patients.

Physiologically, sST2 is a circulating isoform of ST2, which may antagonize the effect of IL-33 mediated by the ST2L by functioning as a decoy receptor [11,38]. Enhanced release of sST2 in peripheral circulating during acute myocardial ischemia further attenuates the cardioprotective effects of the IL-33/ST2L system, which finally contributes to the vulnerability of the patients to cardiac dysfunction and related adverse clinical outcomes [38]. These may be the molecular basis for the prognostic role of sST2 concentration in ACS patients.

Our study has strengths such as including fully adjusted results of the studies, with analyses of both the short-term and long-term prognostic efficacies of sST2 concentrations, and with analyses of sST2 concentrations presented in different variable patterns. However, our study also has limitations, which should be noticed when interpreting the results. First, the number of the included studies in each stratum of the meta-analysis is relatively small, which prevented us from analyzing the sources of heterogeneities that was detected for some outcomes. Second, we did not have individual patient data of the included follow-up studies. Based on the data of study-level, we were unable to determine whether the prognostic value of sST2 concentration differs in patients with STEMI and NSTE-ACS, in the male and the female, and in those with and without cardiac dysfunction. Future studies with large sample sizes are needed to answer these questions. Moreover, as a meta-analysis of observational studies, we could not exclude the chance that some residual factors may confound the association between sST2 concentration and poor prognosis in ACS patients. In addition, the study by Kohli et al. [18] had much larger sample size than others, and for some outcomes, the results seem to be mainly driven by this study, which may lead to bias. However, sensitive analyses by omitting this study retrieved similar results. Finally, different measurement methods for sST2 concentration were applied in the included studies, of which, the Presage assay has been proved to be of better precision than others [36,39]. These may also lead to the heterogeneity of the meta-analysis.

In conclusion, higher sST2 concentration at baseline predicts poor clinical outcome in ACS patients. Current findings support the incorporation of measuring circulating sST2 concentration in clinical practice for risk stratification and decision making in ACS patients.

## Abbreviations

ACS: acute coronary syndrome
BNP: B-type natriuretic peptide
CI: confidence interval
GRACE: Global Registry of Acute Cardiac Events
HF: heart failure
IL-33: interleukin 33
MACE: major adverse cardiovascular event
NSTE-ACS: non-ST segment elevated ACS
RR: risk ratio
sST2: soluble suppression of tumorigenicity-2
STEMI: ST segment elevated myocardial infarction
ST2L: transmembrane isoform of ST2
TIMI: thrombolysis in myocardial infarction
